# Clinical, endoscopic, and pathological features of synchronous multiple early-stage colorectal cancer: a two-center retrospective case–control study

**DOI:** 10.3389/fmed.2026.1929117

**Published:** 2026-10-21

**Authors:** Ranran Zeng, Xingting Guo, Jing Ning, Xiangchun Lin, Jing Zhang, Fang Gu, Xun Liu, Qiao Meng, Hailong Li, Yuxin Zhang, Shigang Ding

**Affiliations:** 1 Department of Gastroenterology, Peking University Third Hospital, Beijing, China; 2 Department of Gastroenterology and Endoscopy Center, Peking University International Hospital, Beijing, China

**Keywords:** colorectal cancer, disease characteristics, early diagnosis, risk prediction model, synchronous multiple primary colorectal cancer

## Abstract

**Objectives:**

To characterize the clinical, endoscopic, and pathological features of synchronous multiple early-stage colorectal cancer (sMECRC) and develop a prediction model to identify patients at risk of synchronous lesions at index colonoscopy.

**Methods:**

We retrospectively analyzed 1,081 consecutive patients with early-stage colorectal cancer who underwent endoscopic treatment at Peking University Third Hospital (2013–2024; development cohort) and 161 patients treated at Peking University International Hospital (2016–2024; external validation cohort). A prediction model was developed using Firth penalized logistic regression, internally validated using 1,000 bootstrap resamples, and externally validated. Sensitivity analyses were also performed.

**Results:**

Of the 1,081 patients in the development cohort, 47 (4.3%) had sMECRC. Compared with patients with a single lesion, those with sMECRC had more concomitant adenomatous polyps (median, 5 vs. 2), larger index lesions (2.50 vs. 2.00 cm), a higher rate of FOB positivity (72.2% vs. 46.5%), and a higher frequency of index lesions in the descending colon (17.0% vs. 4.7%). Independent predictors of sMECRC were positive FOB [odds ratio [OR], 2.441; 95% confidence interval [CI], 1.157–5.150], number of adenomatous polyps (OR, 1.166 per polyp; 95% CI, 1.094–1.244), index lesion size (OR, 1.247 per cm; 95% CI, 1.099–1.416), and descending colon location (OR, 4.411; 95% CI, 1.627–11.959). The model showed moderate-to-good discrimination in the development cohort [apparent area under the curve (AUC),0.767;bootstrap optimism-corrected AUC, 0.748] and had a Brier score of 0.040, although calibration was suboptimal. External validation in an independent cohort of 161 patients, including 16 with sMECRC, confirmed good discrimination (AUC, 0.864; 95% CI, 0.778–0.950), with improved calibration after intercept-and-slope recalibration. Significant correlations were found between index and second lesions for diameter, Paris classification, depressed morphology, depth of invasion, and histological differentiation.

**Conclusion:**

FOB positivity, a greater adenomatous polyp burden, larger index lesion size, and an index lesion located in the descending colon independently predicted sMECRC.Using routine clinical variables, the model exhibited moderate to good discrimination in the development cohort and preserved good discrimination on external validation, but required recalibration for reliable absolute risk estimates. Concordance between the characteristics of index and second lesions may help guide more targeted inspection of the remaining colon.

## Highlights

### Established knowledge of the subject

- Early detection is essential for improving outcomes in colorectal cancer (CRC). Synchronous multiple early-stage colorectal cancer (sMECRC), defined as the presence of two or more independent early-stage CRC lesions, poses additional challenges for complete detection and endoscopic management. However, the clinical and endoscopic characteristics of sMECRC remain insufficiently characterized.
- Evidence regarding risk factors for sMECRC is limited, and no established clinical prediction model is currently available to estimate the risk of synchronous multiple early-stage lesions at index colonoscopy.

### Significant and/or new findings of this study

- Positive fecal occult blood, larger index lesion size, a greater number of concomitant adenomatous polyps, and an index lesion located in the descending colon were independently associated with sMECRC. A Firth penalized logistic regression model incorporating these four routinely available variables achieved an apparent area under the curve (AUC) of 0.767 [95% confidence interval (CI), 0.699–0.834] and a bootstrap optimism-corrected AUC of 0.748 (95% CI, 0.669–0.828) in the development cohort. In the independent external cohort, the model retained good discrimination (AUC, 0.864; 95% CI, 0.778–0.950), although calibration was suboptimal and improved after intercept-and-slope recalibration. These findings support the potential utility of the model for risk stratification; however, prospective validation and recalibration in different clinical settings are required before clinical implementation.
- Among patients with sMECRC, significant correlations were observed between index and second lesions in lesion size, endoscopic morphology, invasion depth, and histological differentiation, with the strongest association observed for depressed morphology. These findings indicate that the endoscopic and pathological characteristics of the index lesion may provide useful clues for identifying synchronous lesions during systematic inspection of the remaining colon.

## 1 Introduction

Colorectal cancer (CRC) is the third most common malignancy worldwide and ranks second among causes of cancer-related mortality (1–3). The high mortality associated with CRC is largely attributable to the substantial proportion of patients diagnosed at an advanced stage. Early-stage CRC (ECRC) is often asymptomatic or associated with only mild symptoms, which may be overlooked by patients. Therefore, early detection and diagnosis of CRC are crucial for improving patient survival and quality of life. ECRC is defined as a tumor with invasion limited to the mucosa (pTis) or submucosa (pT1), irrespective of tumor size or lymph node metastasis (4, 5). The 5-year overall survival (OS) rate after endoscopic resection for ECRC exceeds 90%, with a recurrence rate below 5%, indicating a favorable prognosis (6). Thus, early diagnosis and treatment can significantly reduce CRC mortality. Although most CRC cases present as a single lesion, multiple primary CRC (MPCRC) involves two or more independent lesions occurring either synchronously or metachronously. CRC can be categorized as hereditary or sporadic (7), with sporadic cases accounting for 70%–90% of cases. Sporadic CRC is driven mainly by somatic mutations or environmental factors and lacks a clear hereditary background or family history (8). Clinically, many patients with sporadic CRC present with multiple cancerous lesions, which may manifest as multiple ECRC (MECRC) at an early stage. Undiagnosed synchronous MECRC (sMECRC) may subsequently present as advanced metachronous or interval cancer. According to the latest European Society of Gastrointestinal Endoscopy guidelines, follow-up after endoscopic resection of ECRC aims to detect new or previously undiagnosed tumors (synchronous or metachronous), as well as local residual lesions and recurrences. However, research on sMECRC remains limited, and clinical risk assessment models based on lesion characteristics are lacking. Herein, we compared the clinical, endoscopic, and pathological features of sMECRC and solitary ECRC (SECRC) and examined the associations between index and secondary lesions in patients with sMECRC. We aimed to increase clinicians' awareness of sMECRC, improve its detection, and reduce missed diagnoses. Additionally, we developed a risk assessment model based on clinical risk factors to enable risk stratification of patients with ECRC. This model is crucial for identifying high-risk patients, improving screening efficiency, supporting risk-stratified management of patients with ECRC after treatment, and establishing individualized follow-up strategies.

The published literature on this topic remains fragmented. Synchronous CRC (SCRC) is present at the time of the index examination, and its detection therefore depends on the completeness of the initial colonoscopy. Studies from both Western and Asian populations indicate that synchronous lesions occur in a substantial proportion of patients with colorectal neoplasia and that some are missed during the initial examination (9–12). SCRC, MPCRC, and multifocal colorectal neoplasia are overlapping but distinct concepts, and inconsistent use of these terms has hindered comparisons across studies (13–15). The underlying biological mechanisms are also heterogeneous. Chromosomal instability, microsatellite instability, and aberrant DNA methylation have each been implicated, while field cancerization of normal-appearing mucosa is increasingly recognized as a potential mechanism underlying the development of multiple independent lesions (13, 15–18).

Existing prediction tools for colorectal neoplasia have mainly been developed to estimate the risk of harboring any colorectal neoplasia in screening or symptomatic populations, the risk of metachronous adenoma or cancer after polypectomy, or survival after a diagnosis of CRC, and their reported discrimination has generally been modest [area under the curve (AUC), approximately 0.61–0.70] (10, 19, 20). However, none of these tools addresses the question that arises once an early-stage cancer has been detected endoscopically: whether the patient harbors additional synchronous early-stage lesions. The present model was therefore developed for a different clinical target—the probability of multiple lesions at the index procedure—using exclusively information available before the remaining lesions were identified and was tested in an independent cohort.

## 2 Methods

### 2.1 Study participants

Clinical data were retrospectively and consecutively collected from patients with pathologically confirmed ECRC who underwent endoscopic treatment, including endoscopic mucosal resection (EMR), endoscopic submucosal dissection (ESD), or cold snare polypectomy (CSP), at the Department of Gastroenterology, Peking University Third Hospital between January 2013 and December 2024. Similarly, data were collected from patients with ECRC who underwent the same types of endoscopic treatment at Peking University International Hospital between January 2016 and December 2024 to establish an independent external validation cohort. In both cohorts, patients were consecutively enrolled, and no patient was excluded based on treatment preference; the only reasons for exclusion were the prespecified criteria listed below. This study was approved by the Medical Science Research Ethics Committee of Peking University Third Hospital (IRB No. M2022769) and the Biomedical Ethics Committee of Peking University International Hospital (IRB No. YJ2018-050).

### 2.2 Inclusion criteria

The sMECRC group (experimental group) met the diagnostic criteria for sMECRCs proposed by Moertel (21): ① Patients had two or more primary cancer foci within the colorectum, including synchronous cancers (those detected within 6 months after identification of the first primary cancer). ② Each lesion was pathologically confirmed as ECRC (stage pTis or pT1). Tumor invasion confined to the mucosa was classified as intramucosal cancer (stage pTis), whereas invasion into the submucosa without involvement of the muscularis propria was classified as submucosal cancer (stage pT1). Based on the depth of invasion, stage pT1 was further subdivided into pT1a (SM1; submucosal invasion depth ≤ 1,000 µm, superficial layer) and pT1b (SM2; submucosal invasion depth > 1,000 µm, deep layer) (22, 23). ③ All lesions were independent and separated by normal mucosa; lesions resulting from metastasis, direct invasion, or postoperative residual or recurrent disease were excluded.

Following the criteria of Warren and Gates (9, 24), the index (reference) lesion was defined as the first lesion identified during the index colonoscopy. When two or more lesions were detected simultaneously, the largest lesion observed endoscopically was designated as the index lesion, and the largest of the remaining lesions was designated as the secondary lesion. This operational definition was intentionally selected because it relies solely on information available during the index endoscopy. Ranking lesions by invasion depth or final pathological measurements can only be performed after pathological assessment of all resected lesions and could therefore introduce incorporation bias if used to define the exposure when predicting the presence of additional lesions. For the same reason, all candidate predictors included in the model were variables available before complete lesion ascertainment. Characterization of the paired features of the index and secondary lesions was intended to guide the endoscopic detection of additional (secondary) lesions. Notably, this designation was based on endoscopic size rather than final pathological findings or anatomical order and may therefore have influenced lesion-level comparisons. Accordingly, the paired index–secondary lesion correlation analyses should be interpreted in the context of this operational definition, and the potential for classification bias is acknowledged in the Limitations.

The SECRC (control) group included patients with a single pathologically confirmed ECRC lesion that met the aforementioned diagnostic criteria. For the primary analysis, the model outcome was restricted to sMECRC, defined as two or more independent early-stage (pTis/pT1) lesions detected during the index colonoscopy. Patients with metachronous MECRC were excluded from the modeling cohort and are described separately. Because all model predictors [fecal occult blood (FOB) result, number of concomitant adenomatous polyps, and index lesion location and size] were recorded at the time of the index colonoscopy and were available before complete lesion ascertainment, the model was designed as a diagnostic model for identifying additional synchronous lesions rather than as a prognostic model for predicting future metachronous lesions.

### 2.3 Exclusion criteria

The exclusion criteria were as follows: ① age ≤ 18 years; ② confirmed or suspected hereditary CRC, including familial adenomatous polyposis (FAP), Lynch syndrome, or Peutz–Jeghers syndrome (PJS); ③ inflammatory bowel disease (IBD), including ulcerative colitis (UC) or Crohn's disease (CD); ④ advanced CRC or a history of colorectal resection; ⑤ history of multiple primary malignancies in nonintestinal organs; ⑥ inadequate bowel preparation, defined as a total Boston Bowel Preparation Scale score of <6 or a score of <2 for any intestinal segment (25); ⑦ incomplete clinical, pathological, or endoscopic data; and ⑧ metachronous cancers occurring more than 6 months after the first primary cancer.

### 2.4 Patient selection

For the development cohort at Peking University Third Hospital (2013–2024), 1,128 patients with ECRC underwent endoscopic treatment. Of these, 1,067 had single lesions and 61 had multiple lesions. After applying the prespecified exclusion criteria, 33 patients were excluded from the single-lesion group (1 with advanced CRC, 4 with FAP, 6 with a history of colectomy, 1 with scar recurrence, 20 with incomplete data, and 1 with synchronous early gastric cancer), and 11 patients were excluded from the multiple-lesion group (3 with FAP, 5 with advanced CRC, 1 with Crohn's disease, 1 with a history of colectomy, and 1 with incomplete data). The final development cohort comprised 1,081 patients, including 1,034 with SECRC and 47 with sMECRC (Figure 1). Additionally, three patients developed metachronous MECRC more than 6 months after the index examination. Because the primary outcome of this study was defined as synchronous disease, these three patients were excluded from the modeling cohort and are described separately. For the external validation cohort at Peking University International Hospital (2016–2024), 161 patients were independently enrolled using the same inclusion and exclusion criteria, including 145 with SECRC and 16 with sMECRC.

**Figure 1:**
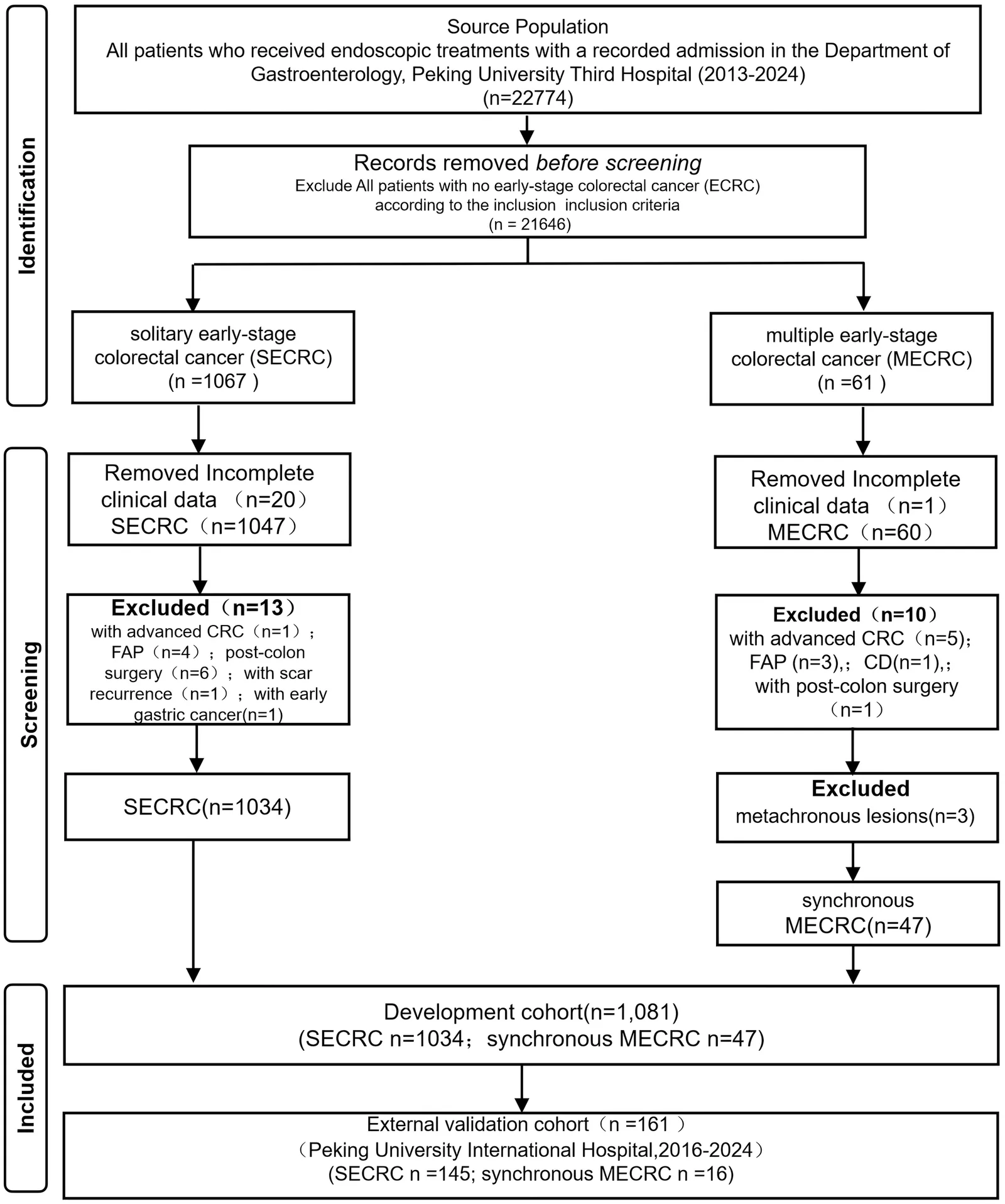
Flowchart of patient selection for the development and external validation cohorts. The primary outcome was synchronous MECRC (sMECRC), defined as two or more independent early-stage (pTis/pT1) lesions detected during the index colonoscopy.

### 2.5 Data collection

Clinical and demographic data were collected from electronic medical records, including age, sex, height, weight, smoking history, alcohol consumption, family history of cancer, comorbidities, recent antibiotic exposure, and presenting symptoms. Body mass index (BMI) was calculated as weight in kilograms divided by height in meters squared (kg/m^2^). Smoking history included smoking duration and smoking index. The smoking index was calculated as the number of cigarettes smoked per day multiplied by the number of years of smoking. For analyses using pack-years, smoking exposure was calculated by dividing the number of cigarettes smoked per day by 20 and multiplying by the duration of smoking in years. Heavy smoking was defined as ≥20 pack-years, consistent with the classification used in previous epidemiological studies of CRC and other cancers (26). Alcohol consumption was recorded based on the available clinical history. Family history of cancer was recorded, with particular attention to CRC in first-degree relatives. Comorbidities included hypertension, diabetes mellitus, coronary heart disease, and liver cirrhosis. Antibiotic exposure within 1 month before endoscopic or surgical treatment was also recorded. Presenting symptoms included abdominal pain, hematochezia, and abdominal distension.

Laboratory data included complete blood count parameters, particularly hemoglobin (HGB); routine stool examination, including FOB; serum biochemical parameters, particularly albumin (Alb); and tumor markers, including carcinoembryonic antigen (CEA) and carbohydrate antigen 19-9 (CA19-9).

The candidate model variables were complete except for FOB results. FOB results were missing for 128 of 1,081 patients (11.84%) in the development cohort and 42 of 161 patients (26.09%) in the external validation cohort. CEA, CA19-9, and serum albumin each had <5% missing data. FOB testing was not routinely required before endoscopic treatment at either participating center. The missingness of FOB results was not completely random, as missing data were more frequent among patients with sMECRC than among those with SECRC (23.4% vs. 11.3%). To further characterize the missing-data mechanism, patients with missing FOB results were compared with those with available results across clinical, endoscopic, and pathological variables (Supplementary Table 4).

Patients with missing FOB values were retained in the descriptive analyses, and the number of missing observations is explicitly reported in Table 1. For primary model development, missing FOB values were handled using multiple imputation by chained equations, with 20 imputed datasets generated and estimates combined according to Rubin's rules. The development and external validation cohorts were imputed separately to avoid information leakage between cohorts and preserve the original cohort structure. Complete-case and missing-indicator analyses were performed as sensitivity analyses.

**Table 1:** Clinical characteristics of patients with synchronous multiple early-stage colorectal cancer (sMECRC) vs. solitary early-stage colorectal cancer (SECRC).

Characteristic	sMECRC (*N* = 47)	SECRC (*N* = 1,034)	Statistical value	*P*-value
Male, *n* (%)	35 (74.5%)	632 (61.1%)	*χ*^2^ = 3.389	0.066
Age, years, mean ± SD	63.43 ± 10.93	62.23 ± 11.33	*Z* = 0.730 (*W* = 25,826.5)	0.466
Age <50 years, *n* (%)	8 (17.0%)	157 (15.2%)	*χ*^2^ = 0.117	0.732
BMI, kg/m^2^, *n* (%)			▴	0.754
<18.5	1 (2.1%)	37 (3.6%)		
18.5–24	21 (44.7%)	402 (38.9%)		
>24	25 (53.2%)	595 (57.5%)		
Clinical symptoms, *n* (%)	27 (57.4%)	608 (58.8%)	*χ*^2^ = 0.034	0.854
Smoking history, *n* (%)	16 (34.0%)	265 (25.6%)	*χ*^2^ = 1.654	0.198
Smoking duration, years	12.87 ± 17.84	7.80 ± 14.77	*Z* = 1.764 (*W* = 27,991.5)	0.022*
Smoking index, pack-years, *n* (%)			▴	0.025*
<10	31 (66.0%)	815 (78.8%)		
10–20	9 (19.1%)	82 (7.9%)		
>20	7 (14.9%)	137 (13.2%)		
Alcohol consumption, *n* (%)	33 (70.2%)	834 (80.7%)	*χ*^2^ = 3.089	0.079
Family history of cancer, *n* (%)	10 (21.3%)	136 (13.2%)	*χ*^2^ = 2.540	0.111
HGB, g/L, *n* (%)			▴	0.576
<120	41 (87.2%)	907 (87.7%)		
≥120	6 (12.8%)	101 (9.8%)		
Missing	0 (0.0%)	26 (2.5%)		
FOB status, *n* (%)			*χ*^2^ = 14.355	<0.001*
Negative	10 (21.3%)	491 (47.5%)		
Positive	26 (55.3%)	426 (41.2%)		
Missing	11 (23.4%)	117 (11.3%)		
CEA, ng/mL, *n* (%)			▴	0.517
≤5	41 (87.2%)	940 (90.9%)		
>5	4 (8.5%)	57 (5.5%)		
Missing	2 (4.3%)	37 (3.6%)		
CA19-9, U/mL, *n* (%)			▴	0.096
≤39	40 (85.1%)	960 (92.8%)		
>39	3 (6.4%)	29 (2.8%)		
Missing	4 (8.5%)	45 (4.4%)		
Alb, g/L, *n* (%)			▴	0.386
>35	45 (95.7%)	1,009 (97.6%)		
≤35	2 (4.3%)	21 (2.0%)		
Missing	0 (0.0%)	4 (0.4%)		

Alb, albumin; BMI, body mass index; CA19-9, carbohydrate antigen 19-9; CEA, carcinoembryonic antigen; FOB, fecal occult blood; HGB, hemoglobin; SD, standard deviation. Missing: missing data counts.

▴ Fisher's exact test was used when the expected cell count was <5. Continuous variables are shown as mean ± SD; non-normally distributed continuous variables were compared using the Wilcoxon rank-sum test, and the *W* statistic was converted to a *Z* value. Percentages within each group were calculated using the total number of patients in that group, including those with missing data.

* *P*-value < 0.05 was considered statistically significant.

Endoscopic variables included lesion location, morphology, size, number of concomitant adenomatous polyps, and other endoscopic features. Lesion location was categorized as the rectum, sigmoid colon, descending colon, or right-sided colon (includes the transverse colon, ascending colon, and cecum). Lesion morphology was classified according to the Paris classification (4) as protruding (0-Ip, 0-Is, and 0-Isp), superficial nonprotruding (0-IIa, 0-IIb, and 0-IIc), or depressed (0-III). Laterally spreading tumors (LSTs) were recorded according to their predominant superficial morphology and classified as 0-IIa when appropriate, including mixed morphological patterns such as 0-IIa + 0-IIc, 0-IIc + 0-IIa, and 0-IIa + 0-Is. Lesion size was determined based on the maximum and minimum diameters of the resected specimen. The number of concomitant adenomatous polyps was recorded separately from the index carcinoma. Additional endoscopic features included erosion, depression, lobulation, leukoplakia, redness, and nodularity.

Pathological variables were assessed according to the Japanese Classification of Colorectal, Appendiceal, and Anal Carcinoma and the relevant criteria of the Japanese Society for Cancer of the Colon and Rectum (JSCCR) (5). The depth of invasion was categorized primarily as intramucosal carcinoma (pTis) or submucosal invasive carcinoma (pT1). For pT1 lesions, the depth of submucosal invasion was further classified according to the Japanese criteria as superficial submucosal invasion (SM1; pT1a) or deep submucosal invasion (SM2; pT1b), with pT1b corresponding to a submucosal invasion depth ≥ 1,000 μm according to the relevant JSCCR criteria (27). The 2019 Vienna Classification was used as a complementary framework for classifying colorectal epithelial neoplasia rather than as the staging system for invasion depth.

Histological assessment included tumor differentiation, histological subtype, tumor budding, and lymphovascular invasion. Histological differentiation was categorized as well-differentiated adenocarcinoma (tub1), moderately differentiated adenocarcinoma (tub2), poorly differentiated adenocarcinoma (por), mucinous adenocarcinoma (muc), or signet-ring cell carcinoma (sig). Poorly differentiated adenocarcinoma was further classified as por1 or por2, where applicable. The well-differentiated category was defined according to the adopted pathological classification and included papillary, tubular, or medullary components, where applicable. Tumor budding was graded as G1–G3 according to the adopted pathological criteria. Lymphovascular invasion, including lymphatic and venous invasion, was recorded as present or absent.

All pathological diagnoses were independently reviewed by two board-certified gastrointestinal pathologists, each with >10 years of experience. The pathologists were blinded to clinical group assignment (sMECRC vs. SECRC). Discordant assessments were resolved through consensus discussion with a third senior gastrointestinal pathologist. A formal interobserver agreement analysis was not performed; however, all cases with potentially ambiguous findings were subjected to consensus review before the final pathological classification was recorded.

### 2.6 Statistical analysis

Statistical analyses were performed using SPSS version 25.0 and R version 4.4.3. Continuous variables with a normal distribution are presented as mean ± standard deviation (SD), and between-group comparisons were performed using the independent-samples *t*-test. Non-normally distributed continuous variables are presented as median (P25, P75), and between-group comparisons were performed using the Mann–Whitney *U* test. Categorical variables are presented as frequency (percentage) and were compared using the *χ*^2^ test or Fisher's exact test, as appropriate. A multivariable prediction model was developed using Firth penalized logistic regression, which reduces bias in coefficient estimates when the number of events is limited. Categorical predictors were entered as dummy variables, with rectal lesions as the reference category, and continuous predictors were entered as continuous variables. To assess multicollinearity among the candidate variables included in the multivariable logistic regression model, the variance inflation factor (VIF) was calculated for each predictor.The final model contained six parameters (FOB, three location dummy variables, longest lesion diameter, and number of concomitant adenomatous polyps), yielding an events-per-variable (EPV) ratio of approximately 7.8 (47 sMECRC events ÷ 6 predictor parameters, excluding the intercept; the corresponding EPV would be 6.7 if the intercept were included), which is below the widely cited minimum criterion of EPV ≥ 10 (28). Given this moderate EPV, Firth penalization was used to reduce small-sample bias, and internal validation using 1,000 bootstrap resamples was performed to estimate optimism-corrected model performance. Internal validation of the model was performed using bootstrap resampling (B = 1,000) with optimism correction, with multiple imputation repeated within each resample. External validation was performed using an independent validation cohort, for which imputation was conducted separately and the coefficients derived from the development cohort were applied without modification. Model discrimination was assessed using the area under the curve (AUC), with 95% confidence intervals (CIs) calculated using the DeLong method and pooled across imputations according to Rubin's rules. Calibration was assessed using the calibration slope and intercept, Brier score, scaled Brier score and Hosmer–Lemeshow goodness-of-fit test. Complete-case and missing-indicator analyses were performed as sensitivity analyses to assess the handling of missing FOB values. To assess the sensitivity of the results to class imbalance, a nested Synthetic Minority Over-sampling Technique for mixed data (SMOTE-NC) analysis was performed. Oversampling was applied only within the training portion of each resampling fold (repeated fivefold cross-validation with 20 repeats; 100 folds in total) and was never applied to the assessment folds or external validation cohort. Missing values within each assessment fold were imputed using a model estimated exclusively from the corresponding training fold, thereby preventing information leakage between the training and assessment data (29). Decision curve analysis (DCA) was performed to quantify the net clinical benefit of the prediction model across a range of clinically relevant decision thresholds. Spearman's correlation coefficient or the contingency coefficient, as appropriate, was used to assess correlations between index and secondary lesions. A *P*-value of <0.05 was considered statistically significant.

## 3 Results

### 3.1 Baseline characteristics

The modeling cohort comprised 1,081 patients, including 1,034 patients with SECRC and 47 (4.3%) with sMECRC. Among the 50 patients with multiple early-stage lesions, 47 (94%) had synchronous cancers and constituted the primary analysis group, whereas the remaining 3 (6%) had metachronous cancers. These three patients were excluded from the modeling cohort and are described separately. Regarding endoscopic treatment, 611 patients underwent EMR, 469 underwent ESD, and 1 underwent CSP. The sMECRC group included 35 men (74.5%:mean age: 63.43 ± 10.93 years), whereas the SECRC group included 632 men (61.1%; mean age: 62.23 ± 11.33 years). There were no significant differences in sex or age between the two groups (*P* > 0.05). The proportion of patients with early-onset disease (aged <50 years) also did not differ significantly between the sMECRC and SECRC groups (17.0% vs. 15.2%, *P* > 0.05) (Table 1).

FOB results were missing for 128 of 1,081 patients (11.8%) in the development cohort and 42 of 161 patients (26.1%) in the external validation cohort. Missing FOB results were more frequent among patients with sMECRC [23.4% (11/47)] than among those with SECRC [11.3% (117/1,034)], indicating that the data were not missing completely at random. A comparison of patients with missing and available FOB results across clinical, endoscopic, and pathological variables is presented in Supplementary Table 4. The two groups differed with respect to smoking history, smoking index, smoking duration, and sMECRC status; these variables were retained in the imputation model described in the Methods.

### 3.2 Univariate analysis

#### 3.2.1 Clinical characteristics

No significant differences were observed between the two groups in BMI, clinical symptoms, smoking history, alcohol consumption, family history of cancer, HGB, Alb, or CEA levels (*P* > 0.05) (Table 1). However, the sMECRC group had a longer smoking duration (*P* = 0.022), higher smoking index (*P* = 0.025), and higher FOB positivity rate (72.2% vs. 46.5%, *P* = 0.004) (Table 1).

#### 3.2.2 Endoscopic and pathological features

The sMECRC group had a significantly greater number of concomitant adenomatous polyps than the SECRC group [5 [3, 8] vs. 2 [1, 5], *P* < 0.001]. The distribution of index lesion locations also differed significantly between the groups, with a higher proportion of index lesions located in the descending colon in the sMECRC group (17.0% vs. 4.7%, *P* = 0.014). Index lesions in the sMECRC group were also significantly larger in both maximum and minimum diameter (both *P* < 0.05; Table 2). However, no significant differences were observed between the groups in other endoscopic or pathological features, including Paris classification, pathological type, invasion depth, tumor budding, and vascular invasion (*P* > 0.05) (Table 2).

**Table 2:** Endoscopic and pathological features of patients with synchronous multiple early-stage colorectal cancer (sMECRC) and single early-stage colorectal cancer (SECRC).

Variable	sMECRC (*n* = 47)	SECRC (*n* = 1,034)	Statistical value	*P* value
Number of adenomatous polyps, median (P25, P75)^a^	5.00 (3.00, 8.00)	2.00 (1.00, 5.00)	*Z* = 5.208	<0.001*
Maximum diameter of index lesion, median (P25, P75)	2.50 (2.00, 3.25)	2.00 (1.50, 3.00)	*Z* = 2.079	0.037*
Minimum diameter of index lesion, median (P25, P75)	2.00 (1.50, 2.75)	1.60 (1.00, 2.50)	*Z* = 2.184	0.028*
Index lesion location, *n* (%)			▴	0.014*
Rectum	11 (23.4%)	301 (29.1%)		
Sigmoid colon	20 (42.6%)	452 (43.7%)		
Descending colon	8 (17.0%)	49 (4.7%)		
Right-sided colon^d^	8 (17.0%)	232 (22.4%)		
Paris classification, *n* (%)			*χ*^2^ = 5.536	0.137
Ip	19 (40.4%)	260 (25.1%)		
Is	5 (10.6%)	151 (14.6%)		
Isp	13 (27.7%)	343 (33.2%)		
IIa^b^	10 (21.3%)	280 (27.1%)		
Histological invasion depth, *n* (%)			▴	0.348
M	34 (72.3%)	806 (77.9%)		
SM1	4 (8.5%)	103 (10.0%)		
SM2	9 (19.1%)	125 (12.1%)		
Histological differentiation, *n* (%)			▴	0.785
Well differentiated	27 (57.4%)	584 (56.5%)		
Moderately differentiated	18 (38.3%)	415 (40.1%)		
Poorly differentiated	2 (4.3%)	35 (3.4%)		
Tumor budding, *n* (%)			▴	0.947
Not assessed^c^	40 (85.1%)	868 (83.9%)		
G1	6 (12.8%)	121 (11.7%)		
G2	1 (2.1%)	24 (2.3%)		
G3	0 (0.0%)	21 (2.0%)		
Blood vessel and nerve invasion, *n* (%)			*χ*^2^ = 0.179	0.672
Negative	43 (91.5%)	973 (94.1%)		
Positive	4 (8.5%)	61 (5.9%)		

a All polyps were pathologically confirmed as adenomatous polyps; hyperplastic and serrated polyps were excluded.

b Includes mixed-type laterally spreading tumors (0-IIa + 0-IIc, 0-IIa + 0-IIc, 0-IIc + 0-IIa, and 0-IIa + 0-Is).

c Tumor budding was not assessed.

d The right-sided colon includes the transverse colon, ascending colon, and cecum.

* *P* < 0.05 was considered statistically significant.

▴ Fisher's exact test was used when the expected cell count was <5.

### 3.3 Multivariable analysis

FOB positivity, number of concomitant adenomatous polyps, index lesion location, and index lesion diameter were entered into a multivariable Firth penalized logistic regression model, with the longest diameter of the index lesion modeled as a continuous variable (per 1-cm increase) and rectal lesions used as the reference category for location. The final model retained FOB positivity [odds ratio (OR), 2.441; 95% CI, 1.157–5.150; *P* = 0.019], number of concomitant adenomatous polyps (per additional polyp: OR, 1.166; 95% CI, 1.094–1.244; *P* < 0.001), longest diameter of the index lesion (per 1-cm increase: OR, 1.247; 95% CI, 1.099–1.416; *P* < 0.001), and an index lesion located in the descending colon (OR, 4.411; 95% CI, 1.627–11.959; *P* = 0.004) as independent risk factors for sMECRC (Table 3). The sigmoid colon (OR, 1.195; 95% CI, 0.544–2.627; *P* = 0.657) and right-sided colon (OR, 1.260; 95% CI, 0.500–3.175; *P* = 0.624) categories did not differ significantly from the rectum, and smoking index was not retained in the final model. The full model coefficients (*β* estimates, standard errors, and model intercept), which allow individual risk scores to be calculated, are provided in Supplementary Table 1.

**Table 3:** Multivariable firth penalized logistic regression analysis of risk factors for synchronous multiple early-stage colorectal cancer in the development cohort after multiple imputation (m=20).

Variable	OR	95% CI	*P*-value
Fecal occult blood positivity	2.441	1.157–5.150	0.019*
Number of concomitant adenomatous polyps (per polyp)	1.166	1.094–1.244	<0.001*
Maximum diameter of the index lesion (per cm)	1.247	1.099–1.416	<0.001*
Index lesion location			
Rectum	1 (reference)	–	–
Sigmoid colon	1.195	0.544–2.627	0.657
Descending colon	4.411	1.627–11.959	0.004*
Right-sided colon	1.260	0.500–3.175	0.624

OR, odds ratio; CI, confidence interval.

* *P* < 0.05 was considered statistically significant.

#### 3.3.1 Prediction model establishment

The Firth penalized logistic regression model achieved an apparent AUC of 0.767 (95% CI, 0.699–0.834) in the development cohort (Figure 2A), with a Brier score of 0.040 (scaled Brier score, 0.029). The Hosmer–Lemeshow test result was significant (*χ*^2^ = 19.01, df = 8, *P* = 0.015), indicating some deviation from perfect calibration, with a calibration slope of 1.047 and an intercept of 0.364 (Figure 3A). To assess the impact of the substantial class imbalance in the development cohort (sMECRC:SECRC = 47:1,034; approximately 1:22), a SMOTE-NC sensitivity analysis was performed, in which oversampling was applied only within the training portion of each resampling fold and never to the assessment fold. The mean AUC across 100 folds was 0.780 (Supplementary Table 3). An improper implementation, in which oversampling was applied before the training–assessment split, yielded a mean AUC of 0.783 (Supplementary Table 3). The difference of 0.003 quantifies the optimistic bias introduced by pre-split oversampling and indicates that the performance of the primary model was not an artifact of the class-imbalanced design. The external validation cohort was not subjected to oversampling and retained its natural class distribution (sMECRC:SECRC = 16:145) to reflect real-world prevalence.

**Figure 2:**
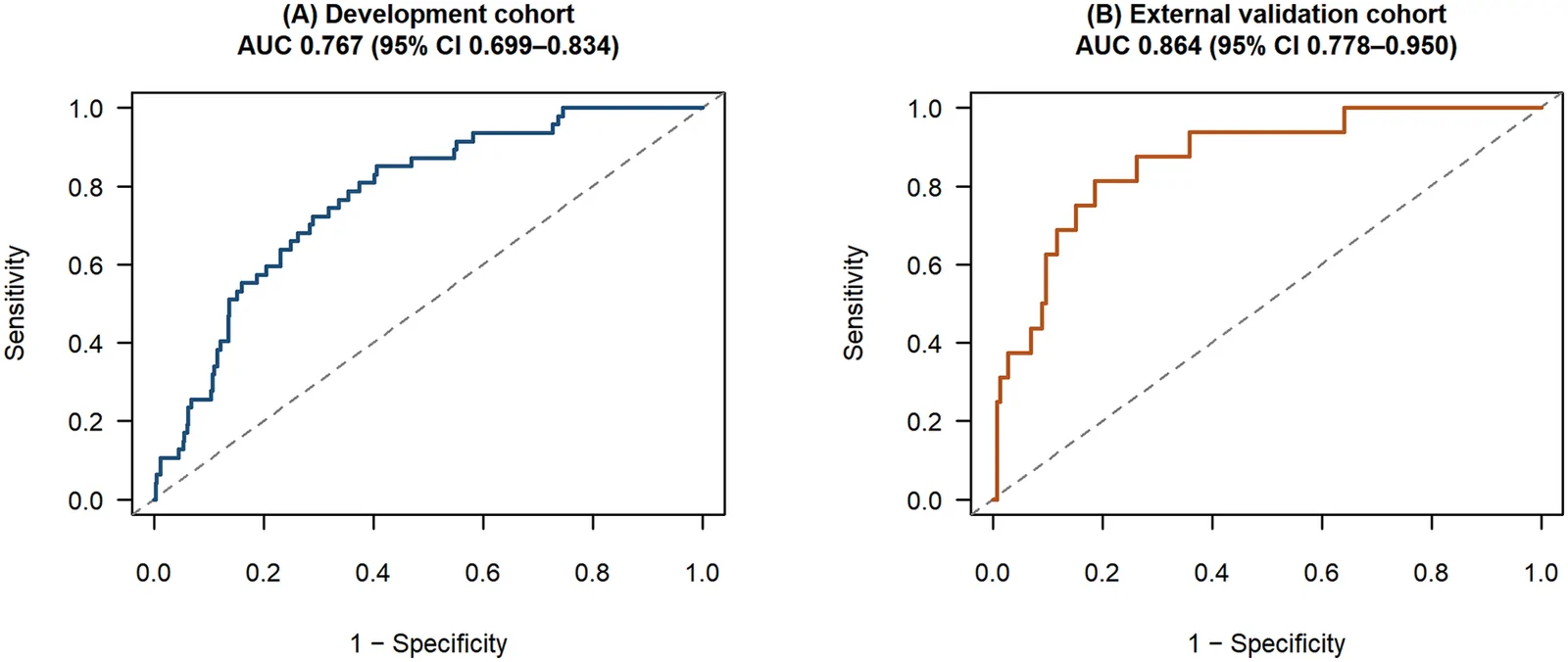
Receiver operating characteristic curves for predicting synchronous multiple early-stage colorectal cancer (sMECRC). **(A)** Development cohort (*n* = 1,081; 47 events); apparent AUC, 0.767 (95% CI, 0.699–0.834). **(B)** Independent external validation cohort (*n* = 161; 16 events); AUC, 0.864 (95% CI, 0.778–0.950). The dashed diagonal line indicates no discrimination. AUC, area under the curve; CI, confidence interval.

**Figure 3:**
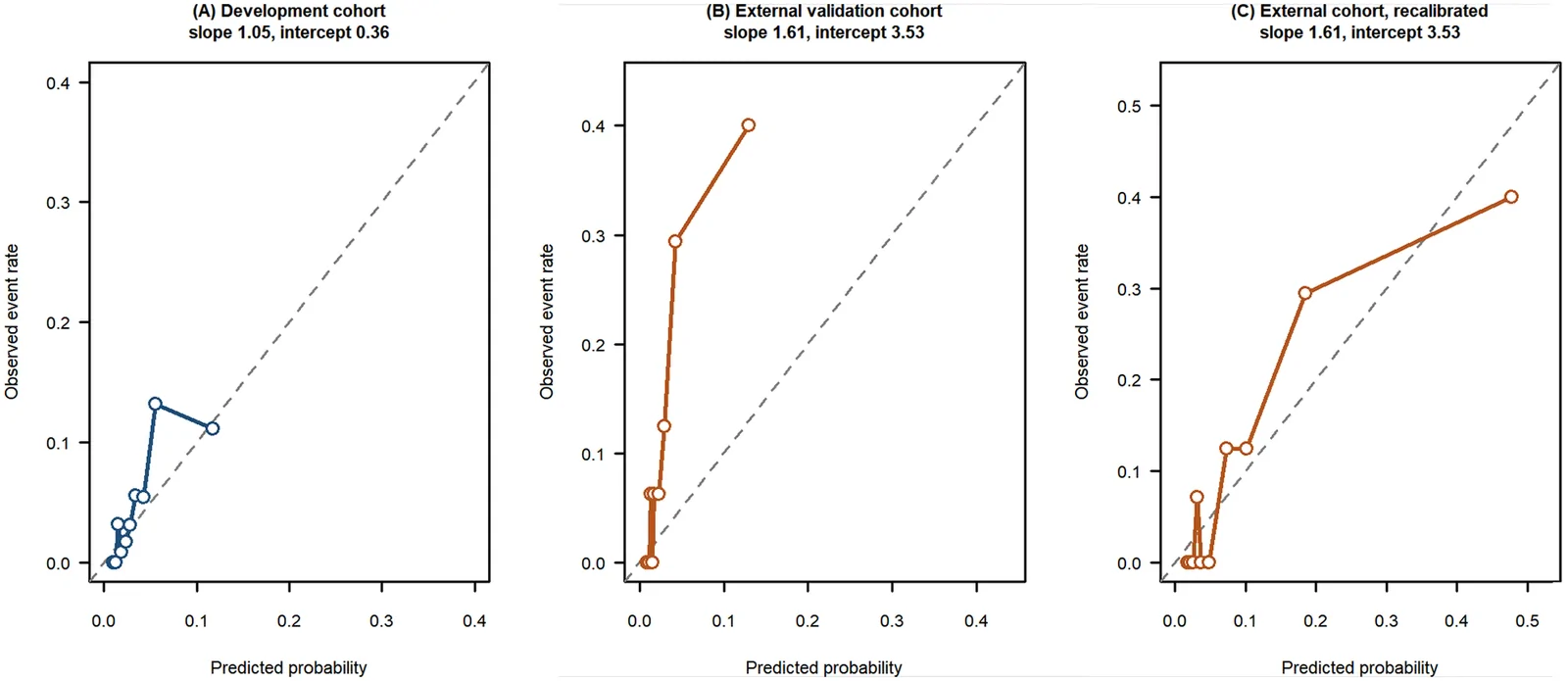
Calibration of the prediction model. **(A)** Development cohort (calibration slope, 1.05; intercept, 0.36; Brier score, 0.040; Hosmer–Lemeshow *P* = 0.015). **(B)** External validation cohort before recalibration (slope 1.61, intercept 3.53, Brier score 0.086). **(C)** External cohort after intercept-and-slope recalibration (slope 1.61, intercept 3.53, Brier score 0.069). Circles show observed event rates by decile of predicted risk; dashed lines indicate perfect calibration.

#### 3.3.2 Internal validation

Internal validation was performed using bootstrap resampling with optimism correction (1,000 resamples, with multiple imputation repeated within each resample). The apparent AUC of 0.767 was corrected to 0.748 (95% CI, 0.669–0.828), with a mean optimism of 0.019, and the optimism-corrected Brier score was 0.041 (scaled Brier score, 0.029). In the development cohort, the calibration slope was 1.047, with an intercept of 0.364, and the Hosmer–Lemeshow test result was significant (*χ*^2^ = 19.01, df = 8, *P* = 0.015) (Figure 3A). Thus, the model showed moderate discrimination but imperfect calibration, with a tendency to underestimate absolute risk. This finding is consistent with the limited number of events (*n* = 47) and the correspondingly wide CIs for the ORs and is the principal reason the model is presented as hypothesis-generating.

#### 3.3.3 External validation

A total of 161 patients met the inclusion and exclusion criteria (sMECRC, *n* = 16; SECRC, *n* = 145) and constituted the independent validation cohort. The baseline characteristics of this cohort are presented in Supplementary Table 2. The cohort retained its natural class distribution to reflect real-world conditions. Model discrimination was maintained (AUC, 0.864; 95% CI, 0.778–0.950; Figure 2B) and was numerically higher than that in the development cohort. However, calibration was unsatisfactory (calibration slope, 1.61; intercept, 3.53; Brier score, 0.086; Hosmer–Lemeshow *χ*^2^ = 47.63, df = 8, *P* < 0.001; Figure 3B and Supplementary Figure 1A), indicating that the model systematically underestimated risk in this population. Intercept-and-slope recalibration performed within the external cohort restored calibration (Brier score, 0.069; Hosmer–Lemeshow *P* = 0.68; calibration slope, 1.000; intercept, 0.000 by construction; Figure 3C and Supplementary Figure 1B) without materially affecting discrimination (AUC, 0.873), as expected with a monotonic transformation. A complete-case analysis restricted to the 119 patients with available FOB results (14 events) yielded an AUC of 0.870 (95% CI, 0.780–0.959) and a Brier score of 0.102, confirming that the imputation strategy did not drive the external validation results. Taken together, these findings indicate that the discriminatory information captured by the model is transportable to a new center, whereas the absolute predicted risks are not, and that center-specific recalibration would be required before the model could be used to inform individual clinical decisions.

### 3.4 Nomogram

A nomogram was constructed from the pooled Firth model using FOB positivity, number of concomitant adenomatous polyps, longest diameter of the index lesion, and index lesion location as predictors (Figure 4). Because the nomogram represents a monotonic linear transformation of the final model, its performance in the development cohort was identical to that of the full model (apparent AUC, 0.767; Brier score, 0.040) and should be interpreted with the same caveats regarding calibration. The corresponding regression equation is provided in Supplementary Table 1, allowing predicted probabilities to be calculated directly.

**Figure 4:**
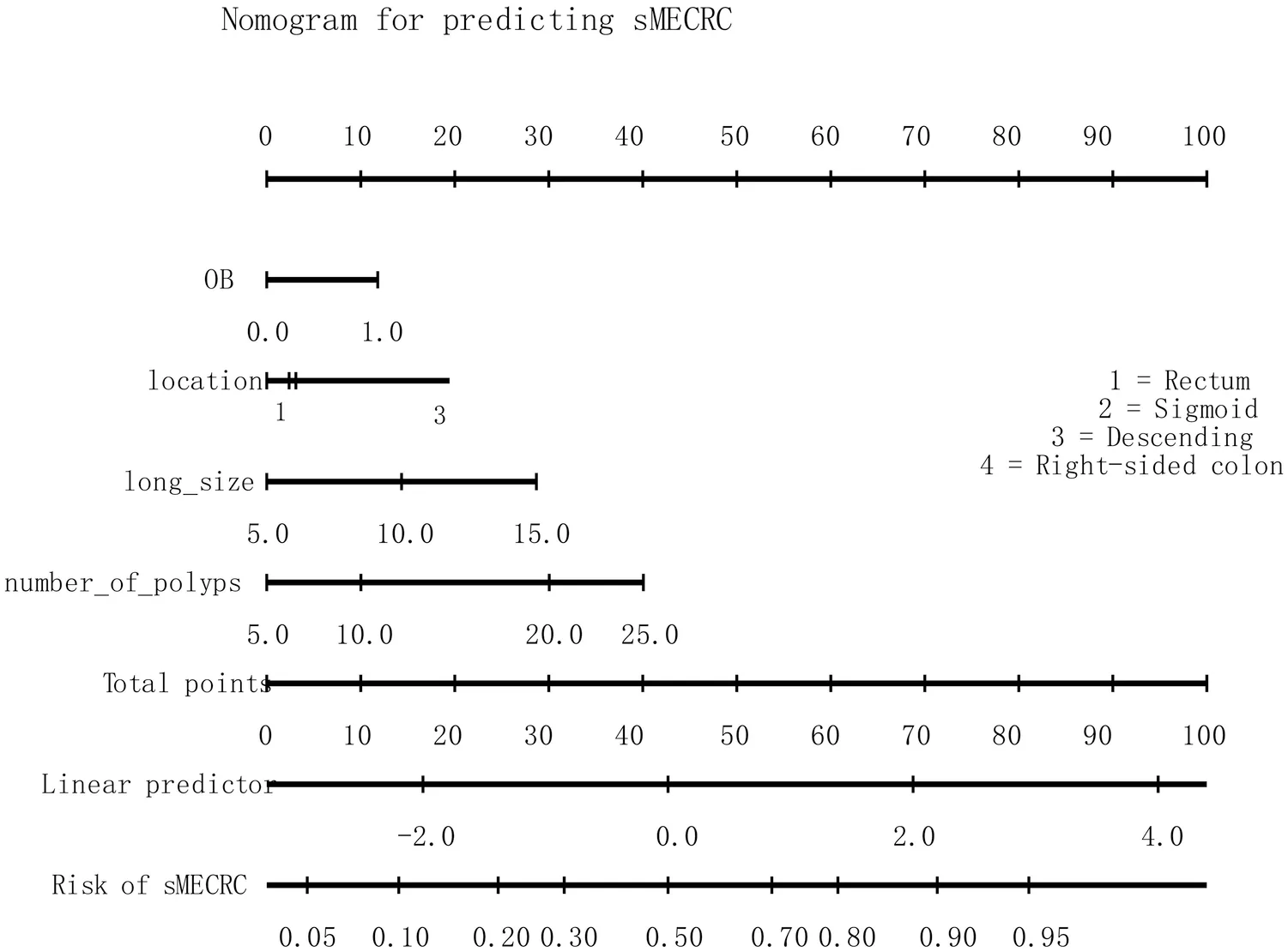
Nomogram for estimating the individual probabilityof synchronous multiple early-stage colorectal cancer (sMECRC). Points are derived from the Firth penalized logistic model. Total points map to the linear predictor and to the predicted probability of sMECRC. Categories (in tick order): location:1 = Rectum; 2 = Sigmoid; 3 = Descending; 4 = Right-sided colon. Tick marks are in category order. OB:fecal occult blood;long_size:longest diameter of the index lesion;number _of_polyps:number of concomitant adenomatous polyps.

### 3.5 DCA

DCA was performed in both cohorts (Figure 5). In the development cohort, the model provided a small but positive net benefit at threshold probabilities of 0.1 and 0.2 (0.0010 and 0.0014, respectively) and only marginally negative net benefits at thresholds of 0.3 and above (−0.0003, −0.0012, and −0.0019 at thresholds of 0.3, 0.4, and 0.5, respectively), whereas the treat-all strategy resulted in substantial net losses throughout (e.g., −0.196 at a threshold of 0.2). In the external validation cohort, the model showed greater net benefits in absolute terms (0.024, 0.005, 0.004, and 0 at thresholds of 0.1, 0.2, 0.3, and 0.5, respectively), compared with −0.126 at a threshold of 0.2 for the treat-all strategy. Across the range of threshold probabilities examined, the model outperformed the treat-all strategy; however, it outperformed the treat-none strategy only at thresholds up to approximately 0.31 in the development cohort and 0.38 in the external validation cohort. Beyond these thresholds, no strategy provided a net benefit. The model provides a small positive net benefit at low threshold probabilities in both cohorts.

**Figure 5:**
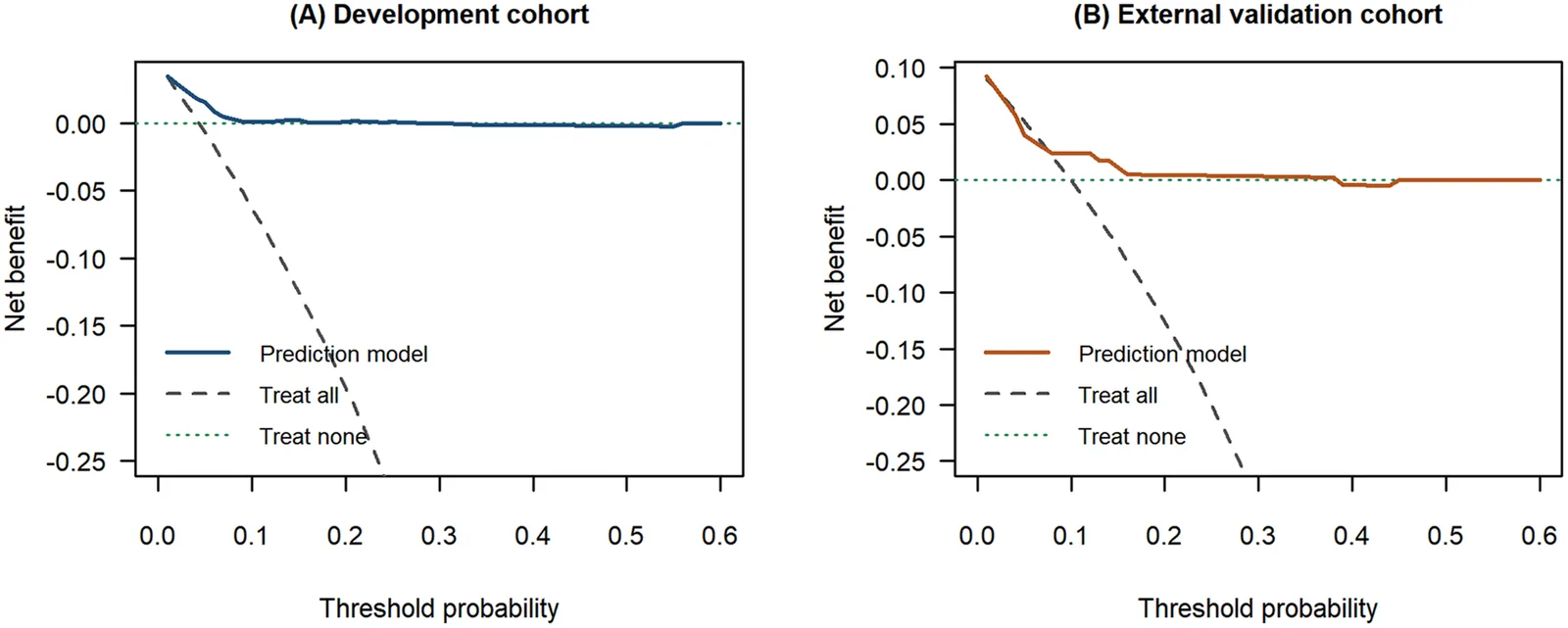
Decision curve analysis of the prediction model. Net benefit is plotted against threshold probability. **(A)** Development cohort. **(B)** External validation cohort. The solid line represents the prediction model, the dashed line represents the treat-all strategy, and the dotted line represents the treat-none strategy.

### 3.6 Correlation between index and second lesions in sMECRC

Among the 47 patients with sMECRC, the maximum diameters of the index and second lesions were positively correlated (Spearman's *ρ* = 0.405, *P* = 0.005; Figure 6), indicating that larger index lesions tended to be accompanied by larger second lesions. Endoscopic morphology according to the Paris classification was also significantly correlated between the two lesions (Spearman's *ρ* = 0.467, *P* < 0.001; Figure 7A). When the index lesion was type Ip or IIa, the second lesion frequently exhibited the same morphology. Depressed morphology showed the strongest correlation (Spearman's *ρ* = 0.884, *P* < 0.001; Figure 7B), with 97.9% concordance between the index and second lesions. Regarding lesion location, 33 of the 47 patients (66%) had both lesions in the left colon (rectum, sigmoid colon, or descending colon), with the sigmoid colon being the most frequent site of co-distribution (11/47, 23.4%). However, the overall correlation between the locations of the two lesions was not statistically significant (Spearman's *ρ* = 0.061, *P* = 0.683). No significant correlations were observed for erosion, lobulation, leukoplakia, or redness (*P* > 0.05). Significant correlations were observed for pathological invasion depth (Spearman's *ρ* = 0.531, *P* < 0.001; Figure 7C) and degree of differentiation (Spearman's *ρ* = 0.491, *P* < 0.001; Figure 7D).

**Figure 6:**
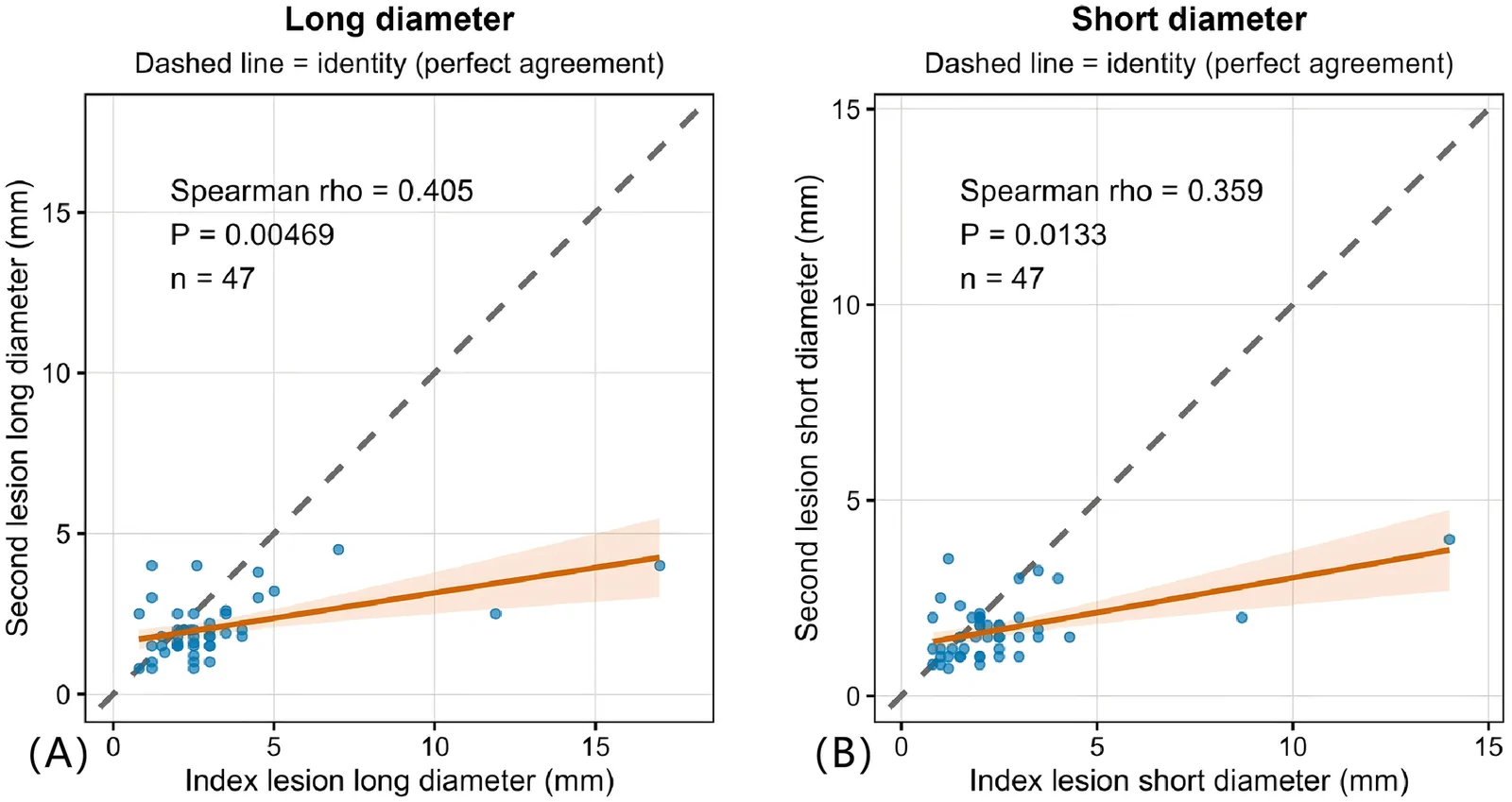
Scatter plots illustrating the correlation between the sizes of the index and second lesions in synchronous multiple early-stage colorectal cancer (sMECRC). **(A)** Relationship between the maximum diameter of the index lesion and that of the second lesion, with a correlation coefficient of Spearman's *ρ* = 0.405, *P* = 0.005). **(B)** Relationship between the minimum diameter of the index lesion and that of the second lesion, with a correlation coefficient of Spearman's *ρ* = 0.359, *P* = 0.013).

**Figure 7:**
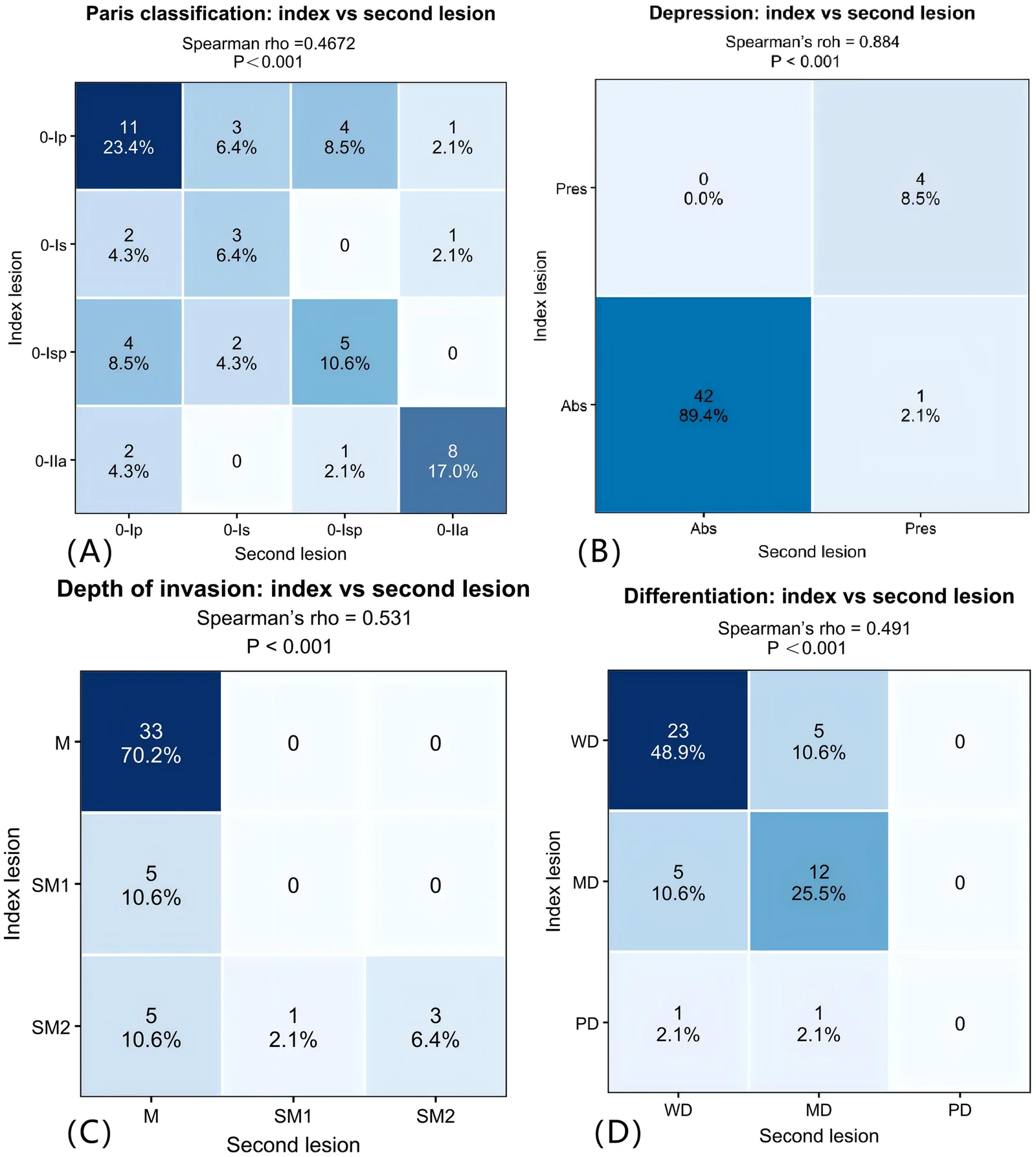
Heat maps illustrating the endoscopic morphological and pathological correlation between the index and second lesions in sMECRC. *ρ* denotes Spearman's rank correlation coefficient; sMECRC, synchronous multiple early-stage colorectal cancer; LST, laterally spreading tumor. **(A)** Association between the index and second lesion on endoscopic morphology according to the Paris classification; a significant correlation existed between the index and second lesion when both are classified as type Ip or type IIa (Spearman's *ρ* = 0.467, *P* < 0.001).0-Isp, mixed; 0-IIa, elevated;0-Ip, pedunculated; 0-Is, sessile. **(B)** A significant correlation existed between the index and second lesion regarding the presence of depressions in the lesions’ endoscopic features (Spearman's *ρ* = 0.884, *P* < 0.001):Abs, absent; Pres, present;. **(C)** Association between the pathological invasion depth of the index and second lesions (Spearman's *ρ* = 0.531, *P* < 0.001). SM1, submucosal invasion depth ≤1,000 µm; SM2, submucosal invasion depth > 1,000 µm. **(D)** Correlation analysis of the pathological differentiation degrees between the index and second lesions (Spearman's *ρ* = 0.491, *P* < 0.001). WD, well-differentiated; MD, moderately differentiated; PD, poorly differentiated.

## 4 Discussion

ECRC is often asymptomatic or associated with only mild symptoms, which may be overlooked by patients. Therefore, early diagnosis and treatment are crucial for reducing CRC mortality. Most CRCs present as single lesions; however, they may also occur as multiple noncontiguous primary lesions, defined as MPCRCs. MPCRC accounts for 5%–10% of all CRCs (2) and can be further classified as synchronous or metachronous CRC (3). The cumulative incidence of MPCRC is approximately 2.9%–3.0% (28) and has reportedly increased in recent years. Studies have shown that the development of MPCRC involves the combined effects of genetic factors, epigenetic alterations, intestinal dysbiosis, and chronic inflammation. In clinical practice, multiple lesions also occur in many patients with sporadic CRC without specific genetic mutations, often during the early stages of disease. Undetected MECRCs may subsequently present as advanced metachronous or interval cancers. Establishing a risk assessment model based on CRC-related factors is important for identifying patients at high risk of MECRCs and improving the effectiveness and efficiency of screening. Although numerous risk assessment models for CRC have been developed, their predictive performance for colorectal neoplasia remains limited, with reported AUCs of 0.61–0.70 (10, 19, 20). Moreover, research on risk assessment models for MECRCs remains limited. In this multicenter, large-sample retrospective study, we characterized the clinical, endoscopic, and pathological features of sMECRC and examined the associations between index and secondary lesions. To the best of our knowledge, no externally validated model for predicting the presence of multiple early-stage lesions at index colonoscopy is currently in routine clinical use, and paired lesion-level analyses have rarely been reported for sMECRC. These findings may facilitate the identification of MECRCs and inform the development of effective screening strategies for high-risk populations.

In this study, patients with sMECRC had a longer smoking duration [median [interquartile range], 0 [0, 30] years; *P* = 0.022] and a higher smoking index (*P* = 0.025). A previous prospective study indicated that both current smoking and greater cumulative smoking exposure are associated with an increased risk of synchronous MPCRC, whereas smoking cessation may reduce this risk. The association between smoking and synchronous MPCRC may be partially mediated by a field effect that promotes molecular alterations, including a high CpG island methylator phenotype (CIMP), high microsatellite instability (MSI), and *BRAF* mutations (30). A prospective cohort study found that compared with solitary CRC, synchronous MPCRC was associated with a higher frequency of *BRAF* mutations, CIMP, and MSI. Recent advances in molecular biology have further identified chromosomal instability, MSI, and aberrant DNA methylation as potential susceptibility factors for synchronous multiple cancers (13, 16).

This study also identified FOB positivity, a greater number of concomitant adenomatous polyps, a larger maximum diameter of the index lesion, and an index lesion located in the descending colon as independent risk factors for sMECRC. We constructed a prediction model with good discriminatory performance, providing new insights into the early identification and accurate diagnosis of sMECRC. In this study, sMECRC accounted for 4.3% of all ECRCs, broadly consistent with previous reports that MPCRCs account for 5%–10% of all CRCs (14). Notably, among the 50 patients with multiple lesions in this study, 47 (94%) had synchronous cancers, whereas the remaining 3 (6%) had metachronous cancers and were included only in the descriptive analysis, indicating that synchronous occurrence was the predominant pattern. This finding may partly reflect advances in endoscopic techniques, as technologies such as high-definition endoscopy and chromoendoscopy have improved the detection of synchronous lesions (31). Compared with patients with SECRC, those with sMECRC showed no significant differences in age or sex distribution. This finding differs from reports of advanced synchronous MPCRCs and synchronous multiple early gastric cancers (SMEGCs), which are more prevalent among older men (32). This discrepancy may be related to differences in disease stage and suggests that multifocal early carcinogenesis in the colorectum and stomach may be influenced by distinct epidemiological factors. Gastric mucosal atrophy with intestinal metaplasia is an important predisposing background for SMEGC, whereas multifocal colorectal carcinogenesis may depend more strongly on factors unrelated to age and sex, such as intestinal dysbiosis and chronic mucosal inflammation (33). Chronic mucosal inflammation and severe intestinal dysbiosis can impair intestinal barrier integrity and disrupt mucosal remodeling, a cascade of events that has been targeted through microbial regulation and therapeutic restoration in chronic colitis models (34). This study further identified an increased number of concomitant adenomatous polyps as an independent risk factor for sMECRC. The sMECRC group had significantly more adenomatous polyps than the control group [median [interquartile range], 5 [3, 8] vs. 2 [1, 5]], suggesting that the detection of multiple colonic adenomas during colonoscopy warrants increased attention to the possibility of multifocal carcinogenesis. This finding supports the “field cancerization” theory (15, 17, 18), whereby long-term exposure of the intestinal mucosa to carcinogenic factors, such as a high-fat diet and intestinal dysbiosis, may induce widespread epithelial alterations that manifest as multifocal adenomatous polyps and increase the risk of multiple primary cancers. Additionally, the FOB positivity rate was significantly higher in patients with sMECRC than in those with SECRC (72.2% vs. 46.5%), and a positive FOB result was independently associated with sMECRC (OR, 2.441). One possible explanation is that multiple lesions increase the extent of colorectal mucosal injury, thereby increasing the likelihood of bleeding and FOB positivity (35). Clinically, FOB testing is widely used for CRC screening. Our findings suggest that patients with ECRC and positive FOB results may warrant careful evaluation for additional synchronous lesions, including thorough inspection of the entire colon and adequate withdrawal time during colonoscopy.

Previous studies have shown that synchronous MPCRCs commonly occur in the same or adjacent intestinal segments, with the rectum, sigmoid colon, and ascending colon being the most frequently involved sites (11, 12). In this study, both solitary and multiple ECRCs were predominantly located in the rectum and sigmoid colon. Moreover, among patients with sMECRC, the index and secondary lesions were most frequently co-located in the sigmoid colon (11/47, 23.4%). However, the overall association between the locations of the index and secondary lesions was not statistically significant. Compared with SECRC, sMECRC was more frequently associated with an index lesion located in the descending colon. This finding may be related to anatomical characteristics of the descending colon, such as its tortuous course, relatively long intestinal segment, slower transit, and prolonged fecal retention (36), as well as local microecological characteristics, including enrichment of potentially harmful bacteria. However, these mechanisms should be regarded as hypotheses rather than established explanations because colonic transit time and regional microbiota composition were not measured in this study. The higher frequency of descending colon index lesions may also reflect differences in the detection, biopsy, or resection of lesions in this segment rather than a genuine biological susceptibility to multiplicity. Dedicated studies incorporating measurements of colonic transit time, mucosal microbiome profiling, and careful documentation of endoscopic detection rates are required to investigate these possibilities (37, 38). These findings suggest that careful inspection for additional synchronous lesions may be warranted when an index lesion is detected in the descending colon, with adequate examination of the remaining colon to minimize the risk of missed lesions. The regression model constructed using these independent risk factors achieved an apparent AUC of 0.767, with a bootstrap optimism-corrected AUC of 0.748, while external validation in an independent cohort yielded an AUC of 0.864. These findings indicate that the model retained good discriminatory performance in the external cohort; however, calibration was suboptimal, indicating that center-specific recalibration would be required before the model could be used to estimate absolute risk for individual patients. Unlike traditional CRC risk models, which have reported AUCs of approximately 0.61–0.70, the present model specifically targets sMECRC and incorporates endoscopy-related variables, including the number of concomitant adenomatous polyps and the location of the index lesion. The nomogram may therefore assist endoscopists in identifying patients with a higher probability of harboring additional synchronous lesions during the index examination. For patients with these risk factors in whom no additional lesions are identified during the initial examination, careful completion of the index colonoscopy and appropriate endoscopic surveillance may be warranted to minimize the risk of missed synchronous or subsequently detected interval lesions.

In this study, we analyzed the associations between index and secondary lesions in sMECRCs and identified several notable features. ① Size association: The maximum and minimum diameters of the index and secondary lesions were significantly correlated (maximum diameter: Spearman's *ρ* = 0.405, *P* = 0.005; minimum diameter: *ρ* = 0.359, *P* = 0.013). This finding suggests that when a large index ECRC lesion is detected, careful examination for additional lesions, including larger secondary lesions, may be warranted. ② Morphological association: Significant correlations were observed between the index and secondary lesions in Paris classification (Spearman's *ρ* = 0.467) and depressed morphology (Spearman's *ρ* = 0.884). Specifically, index lesions classified as type Ip or IIa, including laterally spreading tumors, were frequently accompanied by secondary lesions with similar morphologies. These findings may help guide endoscopic examination: when an LST with a depressed component or a type Ip index lesion is identified, careful inspection for additional lesions with similar morphological characteristics may help reduce the likelihood of missed synchronous lesions. ③ Pathological association: The pathological characteristics of the index and secondary lesions, including invasion depth and degree of differentiation, were significantly correlated, with a correlation coefficient of *ρ* = 0.531 for invasion depth. This association was comparable to that previously reported for SMEGC (*R* = 0.455, *P* = 0.014) (39). Additionally, index lesions in patients with sMECRC showed slightly higher proportions of pT1b invasion and poor differentiation, suggesting a stronger invasive potential (40). Accurate preoperative assessment of lesion morphology and submucosal invasion depth is important when determining the appropriate treatment strategy, including endoscopic resection or surgical intervention. Modern risk-stratified imaging frameworks incorporating high-resolution magnetic resonance imaging and endosonography may improve the accuracy of local staging in ECRC (41). However, because these correlation analyses were based on only 47 patients with sMECRC, the estimates are imprecise and should be considered hypothesis-generating rather than evidence of established biological associations. Therefore, careful pathological evaluation and appropriate follow-up remain important.

This study has several limitations. ① The retrospective design and inclusion of only two centers, with the external validation cohort derived from a single additional hospital and containing only 16 sMECRC events, may have introduced selection and referral biases and limit the generalizability of the findings. ② Because lesion ranking was determined by the order of detection and endoscopic lesion size, designation of the index lesion may have been influenced by the endoscopist's examination sequence. This operational definition was deliberately adopted to avoid incorporation bias; therefore, descriptive comparisons between index and secondary lesions should be interpreted in the context of this definition. ③ A time-to-event framework was not used, and the three patients with metachronous lesions were reported only descriptively. The predictors examined in this study were available at the time of index colonoscopy and are therefore suitable for identifying additional synchronous lesions; however, they cannot be interpreted as predictors of future metachronous or interval cancer, for which a clearly defined index date, surveillance duration, and censoring rules would be required. ④ Only 47 sMECRC events were included, and the model contained six predictor parameters (four predictors, with lesion location contributing three indicator terms, excluding the intercept), resulting in an EPV ratio of approximately 7.8, which is below the conventional minimum threshold of 10. Accordingly, the OR estimates had relatively wide CIs, the optimism-corrected AUC (0.748) was lower than the apparent AUC (0.767), and the model coefficients should be considered exploratory. ⑤ Calibration was imperfect in the development cohort (Hosmer–Lemeshow *P* = 0.015; slope, 1.047; intercept, 0.364) and unsatisfactory in the external cohort (slope, 1.61; intercept, 3.53; *P* < 0.001), although discrimination remained acceptable. Recalibration improved calibration in the external cohort (Brier score, 0.069; *P* = 0.68); however, absolute risk estimates may be center-specific, and the current model therefore cannot reliably estimate an individual patient's absolute risk without local recalibration. ⑥ FOB data were missing for 11.8% of the development cohort and 26.1% of the external cohort, and the missingness was not completely random. Missing FOB data were more frequent among patients with sMECRC (23.4%) than among those with SECRC (11.3%). Multiple imputation was used for the primary analysis, and complete-case and missing-indicator analyses yielded comparable discriminatory performance. However, the missing-indicator term itself was strongly associated with the outcome (OR, 2.61; 95% CI, 1.27–5.39), and the “missing” category should therefore be regarded as an operational indicator rather than a biological risk factor. ⑦ Lesion-level assessment was not supplemented by a formal interobserver agreement analysis, and examinations were performed by multiple endoscopists over a 12-year period, during which endoscopic technology and image quality improved. ⑧ The absence of molecular data (MSI, *BRAF*, *KRAS*, and CIMP status), microbiome data, and multiomics data precludes inferences regarding the biological mechanisms underlying the observed associations and prevents exploration of pathway-specific determinants of multiplicity. Accordingly, the developed model should be regarded as a clinicopathological risk-stratification tool rather than a biological model.

Future research should focus on the following areas: ① conducting prospective multicenter studies to validate the model, increase the number of sMECRC events, and optimize its risk-stratification performance; ② investigating the association between gut microbiota dysbiosis and sMECRC to identify potential predictive biomarkers and further improve the diagnostic performance of the model; ③ elucidating the molecular mechanisms underlying field cancerization and gut microbiota dysbiosis in sMECRC; and ④ developing standardized protocols for endoscopic screening and follow-up of sMECRC based on the observed associations between index and secondary lesions to reduce the risk of missed lesions. To advance beyond current clinicopathological nomograms, future multicenter studies should also integrate multiomics profiling. In particular, a multimodal framework combining the mucosal microbiome, transcriptomics, and digital histology may improve risk stratification and help elucidate the mechanisms underlying field cancerization in multiple colorectal malignancies (42).

## 5 Conclusion

FOB positivity, an index lesion located in the descending colon, a larger maximum diameter of the index lesion, and a greater number of concomitant adenomatous polyps were independently associated with sMECRC. The developed model showed moderate-to-good discrimination, which was retained in an independent external cohort; however, calibration was suboptimal, and local recalibration would be required before absolute risks could be estimated for individual patients. Given the small number of sMECRC events and the absence of prospective validation, the model should currently be considered an exploratory risk-stratification aid rather than a tool for clinical decision-making. Significant associations were observed between the characteristics of index and second sMECRC lesions, which may help guide targeted endoscopic inspection and follow-up, pending confirmation in prospective studies.

## Data Availability

The raw data supporting the conclusions of this article will be made available by the authors, without undue reservation.
