# Supplementary material for: Clinical, endoscopic, and pathological features of synchronous multiple early-stage colorectal cancer: a two-center retrospective case–control study
Source: Front Med (Lausanne). 2026 Oct 21;13:1929117. doi: 10.3389/fmed.2026.1929117 (PMC13736176; doi:10.3389/fmed.2026.1929117)
Supplement: Supplementary file 1 [file Table1.docx]

**Supplementary Table 1: Multivariable model results from the primary analysis using multiple imputation (m = 20) and Firth penalized logistic regression**

| **Variable** | **OR (95% CI)** | ***P*-value** | **β** | **SE** | ***Z*-value** |
| --- | --- | --- | --- | --- | --- |
| Intercept | 0.006 (0.002–0.016) | <0.001 | −5.1283 | 0.5133 | −9.990 |
| FOB positivity | 2.441 (1.157–5.150) | 0.0193 | 0.8925 | 0.3790 | 2.355 |
| Number of adenomatous polyps | 1.166 (1.094–1.244) | <0.001 | 0.1539 | 0.0327 | 4.700 |
| Maximum diameter of index lesion | 1.247 (1.099–1.416) | <0.001 | 0.2211 | 0.0648 | 3.414 |
| Sigmoid colon location | 1.195 (0.544–2.627) | 0.6568 | 0.1783 | 0.4013 | 0.444 |
| Descending colon location | 4.411 (1.627–11.959) | 0.0036 | 1.4840 | 0.5083 | 2.920 |
| Right-sided location | 1.260 (0.500–3.175) | 0.6245 | 0.2307 | 0.4712 | 0.490 |

CI, confidence interval; FOB, fecal occult blood; OR, odds ratio; SE, standard error.

| **Supplementary Table 2:**  **Baseline characteristics of the external validation cohort** | | | | |
| --- | --- | --- | --- | --- |
| **Characteristic** | **sMECRC (N = 16)** | **SECRC (N = 145)** | **Statistical value** | ***P*-value** |
| Male, n (%) | 8 (50.0%) | 93 (64.1%) | χ^2^ = 0.701 | 0.402 |
| Age, years, mean ± SD | 62.06 ± 10.78 | 60.92 ± 11.27 | *t* = −0.401 | 0.693 |
| Smoking index, pack-years, n (%) |  |  | ^▲^ | <0.001* |
| <10 | 8 (50.0%) | 116 (80.0%) |  |  |
| 10–20 | 0 (0.0%) | 10 (6.9%) |  |  |
| >20 | 8 (50.0%) | 19 (13.1%) |  |  |
| FOB status, n (%) |  |  | ^▲^ | 0.012* |
| Negative | 7 (43.8%) | 84 (57.9%) |  |  |
| Positive | 7 (43.8%) | 21 (14.5%) |  |  |
| Missing | 2 (12.5%) | 40 (27.6%) |  |  |
| Number of adenomatous polyps, median (P25, P75) | 6 (4, 11) | 2 (1, 4) | *Z* = −4.345 | <0.001* |
| Maximum diameter of index lesion, median (P25, P75) | 2.50 (1.73, 4.00) | 1.70 (1.20, 2.50) | *Z* = −2.277 | 0.022* |
| Minimum diameter of index lesion, median (P25, P75) | 2.00 (1.42, 3.00) | 1.40 (1.00, 2.00) | *Z* = −2.469 | 0.013* |
| Index lesion location, n (%) |  |  | ^▲^ | 0.006* |
| Rectum | 3 (18.8%) | 50 (34.5%) |  |  |
| Sigmoid colon | 5 (31.2%) | 47 (32.4%) |  |  |
| Descending colon | 5 (31.2%) | 11 (7.6%) |  |  |
| Transverse colon | 1 (6.2%) | 21 (14.5%) |  |  |
| Ascending colon | 0 (0.0%) | 13 (9.0%) |  |  |
| Cecum | 2 (12.5%) | 3 (2.1%) |  |  |
| FOB, fecal occult blood; SD, standard deviation; SECRC, solitary early-stage colorectal cancer; sMECRC, synchronous multiple early-stage colorectal cancer.  **P* < 0.05. ^▲^Fisher’s exact test was used when the expected cell count was <5. | | | | |

**Supplementary Table 3:** Sensitivity analyses of model performance and approaches to handling missing fecal occult blood data

| **Analysis** | **Cohort** | **n (events)** | **AUC (95% CI)** | **Brier score** | **Calibration slope** |
| --- | --- | --- | --- | --- | --- |
| Primary analysis (multiple imputation + Firth) | Development | 1,081 (47) | 0.767 (0.699–0.834) | 0.040 | 1.047 |
|  | External | 161 (16) | 0.864 (0.778–0.950) | 0.086 | 1.609 |
| Complete-case analysis | Development | 953 (36) | 0.802 (0.736–0.867) | 0.035 | 1.023 |
| Missing-indicator analysis | Development | 1,081 (47) | 0.770 (0.709–0.831) | 0.041 | 1.039 |
| SMOTE-NC, nested (leakage-free) | Development (CV) | 1,081 (47) | 0.780 | 0.137 | 0.806 |
| SMOTE-NC before data splitting (leakage control) | Development (CV) | 1,081 (47) | 0.783 | 0.218 | 0.883 |
| Recalibrated external model | External | 161 (16) | 0.873 | 0.069 | 1.000 |

AUC, area under the receiver operating characteristic curve; CI, confidence interval; CV, cross-validation; SMOTE-NC, synthetic minority over-sampling technique for mixed data.

The bootstrap optimism-corrected AUCs were 0.748 for the primary analysis and 0.792 for the complete-case analysis.

**Supplementary Table 4:** Missingness analysis comparing patients with available and missing FOB data

| **Variable** | **Level** | **FOB available** | **FOB missing** | **Statistical value** | ***P*-value** |
| --- | --- | --- | --- | --- | --- |
| **Sex** | Female | 369 (38.7%) | 45 (35.2%) | χ^2^ = 0.606 | 0.436 |
| **Smoking history** | No | 721 (75.7%) | 79 (61.7%) | χ^2^ = 11.394 | <0.001 |
| **Smoking index** | <10 | 758 (79.5%) | 88 (68.8%) | χ^2^ = 8.112 | 0.017 |
| **BMI** | <18.5 | 36 (3.8%) | 2 (1.6%) | Fisher’s exact test | 0.311 |
| **MECRC status** | SECRC | 917 (96.2%) | 117 (91.4%) | χ^2^ = 6.294 | 0.012 |
| **Age** |  | 62.30 ± 11.23 | 62.12 ± 11.90 | *Z* = 0.523; *W* = 517306.0 | 0.601 |
| **Smoking duration, years** |  | 7.62 ± 14.77 | 11.02 ± 15.89 | *Z* = −2.997; *W* = 507895.0 | 0.003 |
| **Number of adenomatous polyps** |  | 3.59 ± 3.40 | 3.22 ± 3.43 | *Z* = 1.755; *W* = 521210.0 | 0.079 |
| **Maximum diameter of index lesion** |  | 2.49 ± 1.52 | 2.76 ± 1.78 | *Z* = −1.549; *W* = 510460.5 | 0.121 |
| **Minimum diameter of index lesion** |  | 1.95 ± 1.22 | 2.10 ± 1.43 | *Z* = −0.368; *W* = 514363.5 | 0.713 |
| **Location** | Rectum | 268 (28.1%) | 44 (34.4%) | χ^2^ = 7.327 | 0.197 |
| **Paris classification** | Ip | 240 (25.2%) | 39 (30.5%) | χ^2^ = 5.931 | 0.115 |
| **Erosion** | No | 907 (95.2%) | 123 (96.1%) | χ^2^ = 0.213 | 0.645 |
| **Depression** | No | 897 (94.1%) | 123 (96.1%) | χ^2^ = 0.822 | 0.364 |
| **Lobulation** | No | 592 (62.1%) | 72 (56.2%) | χ^2^ = 1.641 | 0.200 |
| **Leukoplakia** | No | 748 (78.5%) | 88 (68.8%) | χ^2^ = 6.106 | 0.013 |
| **Redness** | No | 595 (62.4%) | 85 (66.4%) | χ^2^ = 0.763 | 0.382 |
| **Nodularity** | No | 799 (83.8%) | 107 (83.6%) | χ^2^ = 0.005 | 0.943 |
| **Histological invasion depth** | M | 741 (77.8%) | 99 (77.3%) | χ^2^ = 0.577 | 0.749 |
| **Histological differentiation** | Well differentiated | 547 (57.4%) | 64 (50.0%) | Fisher’s exact test | 0.249 |
| **Tumor budding** | No | 801 (84.1%) | 107 (83.6%) | Fisher’s exact test | 0.780 |
| **INF** | No | 893 (93.7%) | 126 (98.4%) | Fisher’s exact test | 0.209 |
| **Vascular invasion** | Negative | 898 (94.2%) | 118 (92.2%) | χ^2^ = 0.832 | 0.362 |

BMI, body mass index; FOB, fecal occult blood; INF, infiltrative growth pattern; MECRC, multiple early-stage colorectal cancer; SECRC, solitary early-stage colorectal cancer.

**Supplementary Figure 1**
